# Mental representation and episodic-like memory of own actions in dogs

**DOI:** 10.1038/s41598-020-67302-0

**Published:** 2020-06-26

**Authors:** Claudia Fugazza, Péter Pongrácz, Ákos Pogány, Rita Lenkei, Ádám Miklósi

**Affiliations:** 1 Department of Ethology, Eötvös Loránd University, Budapest, Hungary; 2 MTA-ELTE Comparative Ethology Research Group, Budapest, Hungary

**Keywords:** Evolution, Psychology

## Abstract

We investigated whether dogs remember their spontaneous past actions relying on episodic-like memory. Dogs were trained to repeat a small set of actions upon request. Then we tested them on their ability to repeat other actions produced by themselves, including actions performed spontaneously in everyday situations. Dogs repeated their own actions after delays ranging from a few seconds to 1 hour, with their performance showing a decay typical of episodic memory. The combined evidence of representing own actions and using episodic-like memory to recall them suggests a far more complex representation of a key feature of the self than previously attributed to dogs. Our method is applicable to various species, paving the way for comparative investigations on the evolution and complexity of self-representation.

## Introduction

The cognitive ability to mentally represent own actions in non-human species is a challenging topic, elusive from the methodological point of view, mainly due to the difficulty of devising methods to test it under ecologically relevant conditions. Dolphins demonstrated the ability to repeat their own actions^1^ and to perform an action that differed from the immediately preceding one^2^. However, as a result of their previous training, they expected the test and therefore, their performance may have relied on a prepared behavioural response. Consequently, it remains unknown whether they formed a specific mental representation of their own past actions.

The ability to mentally represent own actions may constitute one of the main building blocks of the more complex ability to represent the self^1,3^. In this view, self-representation is composed by an array of cognitive traits that may have evolved unequally in different species as a result of different selection processes during their evolution for a similar argument see^4^. Self representation has been widely studied in humans and in other primates e.g.^5–7^, but whether non-human animals represent any aspect of the self remains a controversial topic. Recent findings on metacognition in non-primate species e.g. rats^8^; dolphins^9^ challenge the view that any form of self-representation evolved only in primates. The traditional methodological approach to study self-representation is the mirror self-recognition paradigm that has been applied not only to apes^7^, but also to other species (including elephants^10^; dolphins^11^; magpies^12^ cleaner wrasses^13^). However, this paradigm may reveal only a single aspect of self-representation: visual recognition of one’s own image (cf. mirror-guided body inspection^14^). Moreover, the view of self-representation as an undividable cognitive trait does not disclose its complexity^15,16^, as self-representation extends beyond the visual domain^17^. We argue that, based on evolutionary history and ecology, animal species should differ in the complexity of the mental representation that they may construct about themselves.

Dogs, owing to the social richness of their environment, offer an ideal model species to address these questions. Although dogs can use a mirror to solve a task e.g.^18^, evidence of mirror self-recognition in this species has not been provided and, to date, there is no conclusive evidence about any aspect of self-representation in dogs. We argue that for canines, recognizing own appearance (e.g. color of the fur or presence of a spot on it) may not be of any particular advantage, thus it is unlikely that they evolved representation of their own physical appearence. We suggest that there is a need for a wider array of test paradigms for testing different building blocks of self-representation, and the ecology of the species should be taken into account when selecting the applied method, instead of the current anthropocentric and simplistic approach^19^. Some of the previous studies on dogs made steps along this line of thought. For instance, recently it was found that dogs approach an opening suitable for their body size faster than a too small one, suggesting that they represent their body size^19^. A different study applied a modified version of the mirror-self recognition test focusing on olfaction to test dogs’ ability to recognize their own urine^20^ and showed increased investigation of own modified scent. However, the results should not be considered as conclusive evidence of self-recognition because alternative explanations have not been excluded (e.g. increased investigation may be a ‘surprise’ reaction to a novel feature of a stimulus to which dogs are completely habituated).

Some building blocks of self represention might have contributed to dogs’ success in living in human social groups cf.^21^. Particularly, representation and memorization of own actions may be among the most relevant features of the self for dogs living in the complex and variable social environment provided by humans.

Recently, we provided experimental evidence of episodic-like memory in dogs^22^. Episodic memory is a memory of personal events and episodes in one’s life and, in humans, it is thought to be linked to self-representation because it implies the ability to represent the self in the past e.g.^17,23,24^. This, in turn, may also allow an individual to plan future actions^25^. The existence of episodic-like memory in non-human animals has been investigated using different methodologies that reflect diverse theoretical approaches to its definition. While some authors define episodic memory from the perspective of the content of what is remembered, identifying episodic memory as memory of the *what*, *where* and *when* of an event^26^, other authors define episodic memory based on the mechanism how the past event is encoded and recalled. Accordingly, recall of an event relies on episodic memory when such event was encoded incidentally, that is, without knowing that it had to be remembered^27,28^. Episodic-like memory has been investigated in mammals and birds with both approaches. The research on episodic memory in non-human species was initiated by investigating memory of what, where and when in birds by Clayton and Dickinson 1998^29^ who studied cache-recovery behaviour in scrub jays. Evidence of memory of the object and spatial component of episodic memory (*what* and *where*) has also been documented in one dog (Kaminski 2008)^30^. From the perspective of the type of encoding and recall, episodic memory, defined as memory of events encoded incidentally, has been found in chimpanzees and orangutans^31^, rats^27,32^, pigeons^28^ and dogs^22^.

A crucial methodological criterion of experiments on memory of events that were encoded incidentally is the unexpectedness of the recall test^33^. This ensures that there has been no reason for explicit encoding (i.e. enconding information that is known to be important), hence incidental encoding can be reasonably assumed. As the mental experience of memory retrieval is subjective and, therefore, it is not possible to directly assess it in non-human species, along with other authors e.g.^28,32^, we refer to memory of incidentally encoded events as episodic-like memory.

Here we aimed at testing mental representation and episodic-like memory of own spontaneous actions in dogs. We refer to mental representation as the ability to represent things that are not present to the senses and to episodic-like memory as memory of events (i.e. own actions in our study) enconded incidentally^27,28^. Preliminarily, we tested the ability of dogs trained to repeat their own actions to generalize this rule and repeat also actions not included in the training. These tests resembled the training situation in which, typically, the request to perfom an action was followed by the repeat command. Therefore, after receiving a command to perfom an action, the dogs could expect to be asked to repeat and, consequently, encoding of the perfomed action might have been explicit and the dogs’ performance could have relied on a prepared behavioural response.

As our primary aim was to test whether dogs represent actions performed by themsleves spontaneously in everyday-life contexts (i.e., out of a trainig context) and recall those relying on episodic-like memory, we also tested them in situations when the repeat test was unexpected. These tests were carried out in everyday-life situations that did not resemble training and, importantly, the dogs were not given any command to perfom an action but, instead, their owners apparently ignored them. The (unexpected) repeat command was given as soon as the dog spontaneously perfomed a well-identifiable action (e.g. drank water from its bowl or lied down).

In our previous study on episodic-like memory^22^ the tests were carried out in a training/testing context and the test was preceded by a human demonstration of an action, necessarily. Thus, to prevent dogs from encoding the episodes explicitly, we had to ‘re-train’ them to modify their expectation of the test. It has been argued^34^ that, although evidence of the unexpectedness of the memory assessment was compelling, we cannot be certain that encoding was incidental because, in the training history of the dogs, action demonstrations were reliably followed by an imitation test. Consequently, action demonstrations may have been explicitly encoded, in anticipation of the memory assessment. To address this issue, in the key tests of the current study no previous commands are given to the dogs and the situation does not resemble a training context. This ensures that the test was completely unexpected and encoding of the actions performed must have been undoubtedly incidental^33,35^.

## Results

### Potentially expected tests

We applied a specific training procedure (Repeat training, see supporting information), to teach 10 dogs to reproduce their own actions on command ‘Repeat!’. We assessed the success of the repeat training in a *Baseline repeat test* during which the owner asked his/her dog to perform and then repeat the actions used for the Repeat training. This test was done to verify the success of the training process and to assess the baseline level of success of dogs repeating trained own actions. The dogs (N = 10) successfully repeated their own actions irrespectively of whether those were familiar (*Baseline*) or this was the first time they were asked to repeat those (*Untrained actions repeat*), when they could potentially have expected the repeat test (Likelihood Ratio Test (LRT), effect of Experimental condition: χ^2^_1_ = 2.37, p = 0.124; Table 1). These two tests confirmed, therefore, that our Repeat training (see supporting information) was successful.

**Table 1:** Percentage of total trials in which dogs repeated their own actions in the different tests after various delays.

Test condition	Expected or unexpected test	Delay between action of the dog and repeat command	Successful trials in repeating action
Baseline	Expected	No delay	84.2%
Untrained actions	Expected	No delay	73.3%
Doing nothing	Exepcted	No delay	90%
Clever Hans control	Expected	No delay	100%
‘Who is acting’ test	Expected	No delay	88.3%
Spontaneous action	Unexpected	No delay	70%
		20 s	70%
		1 min	60%
		1 h	30%
Spontaneous object-action	Unexpected	No delay	70%
		20 s	70%
		1 min	50%
		1 h	40%
Spontaneous action control	Unexpected	Repeat command is not given	0%
Spontaneous object-action control	Unxpected	Repeat command is not given	0%
Different word control	Expected	No delay	0
Control for context	Unxpected	20 s	0

To test how flexibly the dogs could generalize the repeat rule, dogs were asked to repeat staying still in a sitting position in the *Doing nothing* test. In this test, 9 (of 10) dogs stayed in their position for at least 5 s (thus repeated ‘staying’ or ‘doing nothing’). The remaining one dog lied down 4 s after the repeat command was given.

We controlled for potential unvoluntary cues driving the preformance of the dogs in the *Clever Hans control* test a test. All 10 dogs repeated their own actions when the command was given by person who did not know the past action of the dog, excluding this explanation for the dogs’ performance.

**Figure 1:**
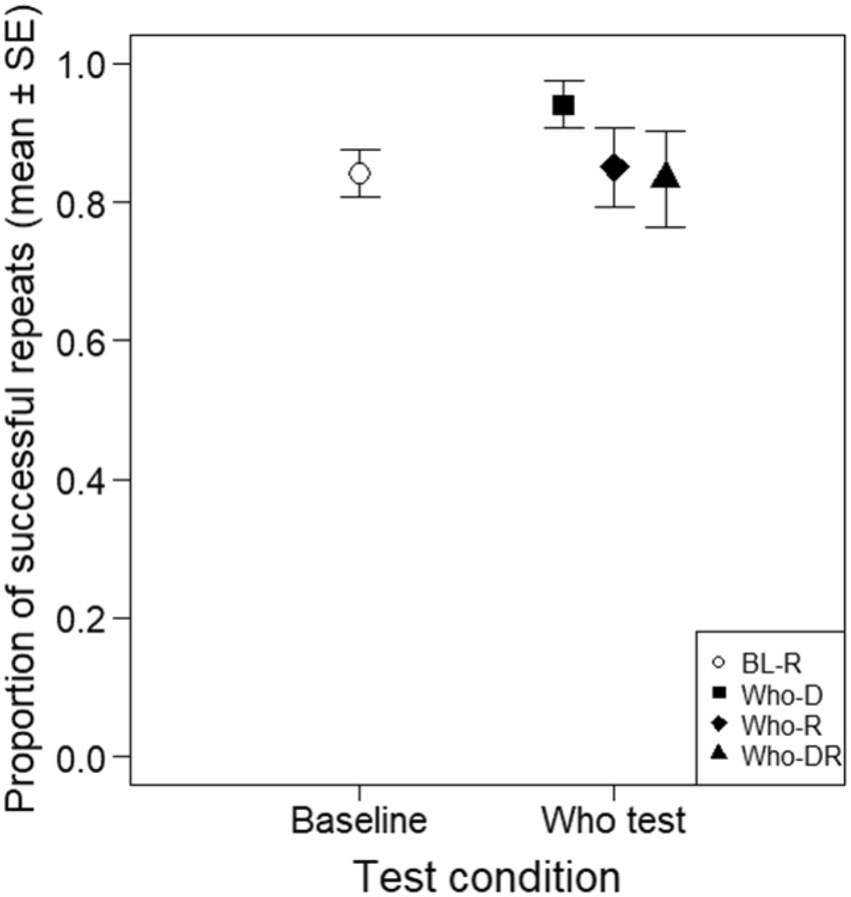
Proportion of successfully repeated own actions or actions demonstrated by the owner in the *Baseline repeat test* (open circle) and in the ‘*Who is acting’* test (filled symbols). The graph shows responses of the 10 dogs in different test conditions: in the Do it trials (Who-D; the owner demonstrated an action and gave the ‘do it’ command); in the repeat trials (Who-R; the owner asked the dog to perform a trained action and then gave the repeat command); and in the Do it + repeat trials (Who-DR; the owner demonstrated an action, gave the ‘do it’ command and, after the dog performed an action - i.e. imitated - gave the repeat command).

In the ‘*Who is acting’ test* the dog was randomly asked to either repeat its own action, or to imitate the action demonstrated by its owner or first imitate an action demonstrated by its owner and then to repeat its own action. The dogs’ performance (88.3% successful trials) did not differ from the Baseline, irrespective of whether the owners issued the repeat command, the Do it command or the repeat command following the ‘Do it’ command - LRT of Experimental condition: χ^2^_1_ = 0.88, p = 0.348 (Fig. 1).

### Unexpected tests

**Figure 2:**
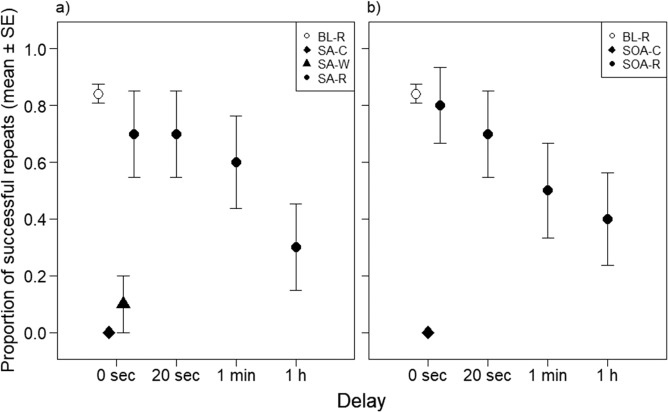
Proportion of successfully repeated actions of 10 dogs in the different experimental conditions, including control tests. Open circles represent the *Baseline condition* (BL-R), in which the dog was asked to repeat actions that were used during the Repeat training. On panel a), success rates during the *Spontaneous action test* (SA-R, black filled circles) and two corresponding control tests: the *Spontaneous action control* (when no command was issued but the dog was let free again in the test area; SA-C, black diamond) and the *Different word control* (when instead of repeat, a different word was said; SA-W, black triangle) are illustrated. On panel b), success rates during the *Spontaneous object-action test* (SOA-R, black filled circles) and the *Spontaneous action control* (when no command was issued; SOA-C, black diamond) are illustrated. In the two Spontaneous repeat tests (SA-R and SOA-R) dogs were tested with different delays (0 sec, 20 sec, 1 min, 1 h) between their spontaneous action and requesting to recall and repeat it.

In the unexpected tests, dogs were able to repeat actions that they performed spontaneously, in everyday situations (*Spontaneous action test* and *Spontaneous object-action test)*. In the *Spontaneous action test*, the repeat command was issued by the owner while sitting on a bench or sofa (i.e. while apparently ignoring the dog that was free in the area) as soon as the dog spontaneously performed a well-identifiable action (e.g., jumped on the sofa, sat, laid on the floor, drank water from its bowl etc.). In the *Spontaneous object-action test*, novel objects were provided that the dog was free to explore (i.e., the objects were on the floor) and the repeat command was issued after an identifiable action with any of the objects was observed. We did not find significant differences between the subjects’ ability to repeat own actions in any of the Spontaneous tests, compared to the *Baseline* (LRT of Experimental condition, *Spontaneous action* vs *Baseline*: χ^2^_1_ = 1.14, p = 0.286; *Spontaneous object-action* vs *Baseline*: χ^2^_1_ = 0.11, p = 0.738; Table 1, Fig. 2). In the *Spontaneous action test* and *Spontaneous object-action test*, 7 of 10 dogs repeated their own actions.

We ensured that dogs would not simply perform a situation-specific action in a given situation (i.e., even if not requested to repeat), by reintroducing the dog in the same context whithout giving the repeat command. In the *Spontaneous action control* and *Spontaneous object-action control* tests none of the dogs repeated the action they had performed previously, thereby excluding that they would always perform those actions in that context. Dogs showed various actions during this test, including sniffing on the floor, licking the owner’s face, lying down and interacting with different objects in the *Spontaneous object-action control*.

In line with the above controls, in the *Different word control* trials, when a different word of no meaning to the dogs was uttered instead of the repeat command, none of the dogs repeated the previously performed action. All dogs except for two, each in one trial, stood in their position for at least 5 s before starting other activities after hearing the meaningless word. One dog lied down and another dog looked at the owner and walked away.

We also tested the dogs by including delays of different lengths (20 s, 1 min, 1 h) between the actions produced spontaneously and the command to repeat in the spontaneous tests. During the delay the dog was taken away from the context where it performed the action and was kept in a crate, thereby limiting the other behaviours that it may spontaneously perform. Dogs’ success decreased gradually with time. With 20 s delay, dogs’ performance was not significantly different from *Baseline* in either test conditions (LRT of effect of delays, 20 s vs *Baseline*, *Spontaneous action test* and *Spontaneous action-object test*, both p > 0.286). When the delay was 1 min, dogs’ performance decreased further (LRT of effect of delays, 1 min delay vs *Baseline*, *Spontaneous action test*: χ^2^_1_ = 3.026, p = 0.082; *Spontaneous object-action test*: χ^2^_1_ = 5.645, p = 0.018). In both test conditions, when tested after the longer delay of 1 h, success to recall own actions was lower than in the *Baseline* (LRT of effect of delays, 1 h delay vs *Baseline*, *Spontaneous action* test: χ^2^_1_ = 13.03, p < 0.001; *Spontaneous object-action test*: χ^2^_1_ = 8.97, p = 0.003; Fig. 1). Specifically, in the *Spontaneous action test* with 20 s delay, 7 of 10 dogs repeated their own actions; with 1 min delay 6 dogs, whereas with 1 h delay 3 of 10 dogs were successful. In the *Spontaneous object action test* with 20 s delay 7 of 10 dogs repeated their own actions; with 1 min delay 5, and with 1 h delay 4 of 10 dogs were successful.

In the *Control for context* test the repeat command was given in a different context, in which none of the dogs (N = 4) repeated their own actions.

## Discussion

We provide experimental evidence of mental representation of own actions and episodic-like memory to recall them, as assessed by the means of unexpected recall tests, revealing dogs’ ability to represent spontaneous actions performed by themselves in the past. Dogs were able to repeat their own actions, irrespectively of whether these were included in the ‘repeat training’ or not. Transfer tests of this kind, in which successful performance on one cognitive task is applied to another, ensure that the subjects learned a rule and not a stimulus-response association^36^. Moreover, dogs were also able to repeat their own actions in tests where the command to repeat was alternated with the command to imitate others’ actions and in trials in which they were asked to ‘stay’ (i.e., ‘do nothing’), thereby showing flexibility in generalizing the ‘repeat rule’ to different situations.

Most importantly, dogs were able to repeat their own actions when the command to repeat was issued unexpectedly, in everyday-life contexts. When trained animals are tested in a test that resembles the training situation, it is difficult to determine whether mental encoding is incidental and the test is indeed unexpected^22^. By testing dogs in everyday-life situations that did not resemble training or testing sitations, we ensured that encoding of their own actions was incidental and, consequently, that remembering those must have involved the using of episodic-like memory. We can, thereby, exclude the possibility that the dogs’ performance relied on a prepared behavioural response, in contrast with previous studies in non-human species that did not rule out this possibility e.g. dolphins^1,2^.

The numerous controls that we ran in this study convincingly exclude that the dogs would always perform those actions in those situations and make it less likely that the prior experience of having performed a given action in that context simply increased the probability of that response due to priming^37^.

The dogs remembered and repeated their own spontaneous actions also after delays of 20 seconds and, in the case of the *Spontaneous action test*, after 1 minute. Some dogs recalled their own actions even after 1 hour. Success in these tests shows that the dogs had formed a mental representation of their own previous action and used it as the basis for performing the same action again, after some delay, when unexpectedly asked to do so. We argue that the combined evidence of mental representation of own spontaneous actions and episodic-like memory to recall them, as assessed by the means of unexpected recall tests, reveals a far more complex representation of a key feature of the self than previously attributed to dogs.

As expected^38^, dogs’ memory of their own actions presented a steep deterioration with increasing delays, showing a decay that is in line with previous studies in which recall recruited episodic memory^22^, further supporting that recall in these tests relied on this type of memory (cf.^22^ and^39^, in which expected/unexpected memory tests were carried out with delays similar to those in the present study). In the test where the context was changed between encoding and recall, dogs were not successful in repeating their own actions. Decreased performance when context is changed between encoding and retrieval has also been found in the case of human episodic memory (e.g.^40,41^. However, for this test we only had the opportunity to test 4 subjects, therefore a relatively small sample size warrants caution when interpreting these findings.

We suggest that the ability to represent and remember own actions relying on episodic memory is an important building block of the more complex ability to represent ‘the self’. Such building block of self-representation may have evolved earlier in evolution and could be more widespread than the more complex conceptual knowledge about the self^42^. Its presence, however, does not imply also the presence of autonoetic consciousness see also^43^. Thus, we do not argue that these results show a fully fledged conceptual knowledge of the self in dogs. We rather argue that representation of own actions, one form of representation of the self, has evolved in this species, that is phylogenetically distant from humans, and may have evolved in a range of other social animals too.

We acknowledge that this study was conducted on a relatively small number of dogs; both the extensive efforts of training the subjects with the two methods applied and testing them in numerous control conditions to exclude alternative explanations limited further subject recruitment. Nevertheless, we argue that our results provide solid evidence to support our conclusions. Our methodological approach has the potential to be applied to other species. This paves the way to new research on the evolution of functionally equivalent abilities, by shedding light on mental self-representation in humans as an array of cognitive traits that may reveal a mosaic evolutionary pattern in different species^44^. These features of the self-representation eventually might have converged and interlocked in humans into a versatile ability to be aware of the self.

## Methods

We applied a specific training procedure (Repeat training, see supporting information), to teach 10 dogs to reproduce their own actions on command ‘Repeat!’. The dogs were also trained to imitate actions demonstrated by a human with the Do as I Do method^45^ (Supporting information). Dogs were tested in the following 12 tests, always starting with the *Baseline test* followed by the other tests in random order, apart from the *Control for context test* (in which only four dogs participated), that was the last test administered. Apart from this last test, each dog participated in every test.

### Potentially expected tests

#### Baseline repeat test

The *Baseline repeat test* consisted of 12 trials during which the owner asked his/her dog to perform and then repeat the six actions used for the Repeat training. At the beginning of each trial, the owner placed his/her dog in front of him/herself, then requested the dog to perform a predetermined action using commands known by the dog. As soon as the dog completed the command, the owner led the dog back to the starting position in front of him/herself and gave the repeat command while looking straight ahead, thus avoiding to give any inadvertent cues. After the dog had performed an action, irrespectively of whether it correctly repeated the previous one or performed something different, the test continued with the next trial, once the owner had repositioned the dog again in the starting position in front of him/her. The order of the six requested actions was semi-randomized so that every action was requested two times within the 12 trials.

#### Untrained actions repeat test

The dog was asked to perform actions it was already trained to perform, but had never been asked to repeat. The procedure was identical to that of the *Baseline repeat test*, but the requested actions differed from those included in the Repeat training. As the already-trained actions varied from dog to dog due to their training history, the type and number of actions used in the test also varied. The number of actions in which the dogs were tested varied from 4 to 8 (mean ± SD = 6.3 ± 0.6) Example of actions included: sit, touch a cone, bark, enter the agility tunnel, jump over a hurdle, spin.

#### Doing nothing test

The owner asked the dog to stay in a sitting position (‘Stay!’, i.e. do not move) using cues known by the dogs and waited for 5 s. Then the owner gave the repeat command. The behaviour of the dog was recorded for the following 20 s. This test consisted of one trial per dog.

#### Clever Hans control

The owner and another person, familiar to the dog, stood back to back. The familiar person attracted the dogs’ attention and called the dog in front of him/her. From this moment on, the owner closed his/her eyes and ears and sang a song loudly in order to ensure that s/he would not hear any noise potentially made by the dog. The familiar person asked the dog to perform a trained action using only visual cues (i.e. gestures). Immediately after, the familiar person touched the owner’s side with hand as an agreed signal to exchange position so that the owner now moved in front of the dog, that typically had returned to its original position after performing the requested action. The owner opened his/her eyes and ears and gave the repeat command without knowing what action the dog had previously performed. This test consisted of one trial per dog.

#### ‘Who is acting’ test

In this test each dog was asked to either repeat its own actions (Repeat trials: R) or to imitate actions demonstrated by its owner (Do it trials: D) in a single 12-trial session. The test procedure in the R-trials was the same as described above for the baseline test. In the D-trials, the owner demonstrated an action, then asked the dog to imitate it (‘Do it!’). We also included trials in which the owner demonstrated an action, asked the dog to imitate and then asked the dog to repeat (Do it + Repeat trials: DR). Note that every DR trial included two actions by the dog: the imitation of the action demonstrated by the owner and then the repetition of the action just performed by itself. The order of the type of trials was semi-randomized and was the same for each dog: R, DR, R, D, R, D, DR, R, DR. The actions demonstrated or requested were those included in the baseline test.

### Unexpected tests

We tested the dogs’ ability to reproduce their own spontaneous actions in everyday-life situations that did not resemble training/testing situations, in absence of a command to perform a specific action.

#### Spontaneous action test

The owner sat on a bench (or chair or sofa) in a place that was familiar to the dog: its house or outdoor area, based on areas suitable for the test that were available for the dog owners and familiar for the dogs. The owner was instructed to type on his/her mobile phone or to read a book, thus apparently ignored the dog that had the opportunity to move freely in the area. As soon as the dog spontaneously preformed a well-identifiable action (e.g., lied down, drank water from a bowl, jumped on a sofa), the owner called the dog in front of him/her and gave the repeat command. The repeat command was always given by the owner while looking straight ahead, in order to avoid inadvertent cues (see also *Clever Hans control above*). To prevent dogs from forming expectations about being tested due to repeated exposure to the tests, we carried out one such trial per dog. These situations were not practiced during training.

#### Spontaneous object-action test

Four objects that were novel for the dog – a wooden statue in shape of an animal, a plush toy, a dog crate and a doll – were placed in a familiar area (house or outdoor area) at 50 cm from each other.

The owner approached the area with the dog unleashed and let the dog to explore freely, while the owner remained passive. As soon as the dog spontaneously preformed a well-identifiable action (e.g., touched an object with its paw, grabbed an object with its mouth), the owner called the dog back to him/herself and gave the repeat command while looking straight ahead. We carried out one such trial per dog and these situations, similarly to the *Spontaneous action test*, were not practiced during training.

In the above two conditions (*Spontaneous action test* and *Spontaneous object action test*), although we did not plan to insert a specific delay between the spontaneous action of the dog and the repeat command, the command was issued 5–15 s after the spontaneous action of the dog (this time was needed for the owner to call the dog back from where the action was performed and to give the repeat command).

#### Spontaneous action control and spontaneous object-action control

In this control condition, after the dog spontaneously performed a well-identifiable action, the owner called it, but did not give the repeat command. Instead, the dog was let free again in the area for 30 s. This was done for both the *Spontaneous action test* and *Spontaneous object-action test*, so that every dog participated in two such trials overall.

#### Different word control and spontaneous action word control

The owner asked the dog to perform a trained action (similarly to the *Untrained actions repeat test*). After the dog had performed the action, instead of giving the repeat command, the owner uttered a word of no meaning for the dog (‘Blue’ or ‘Farfalla’), always looking straight ahead.

This control was also carried out in the *Spontaneous action test* (*Spontaneous action word control*). In this case, after the dog had spontaneously performed an identifiable action, the owner called the dog and said the word of no meaning. This control consisted of two trials for every dog, one with the first action being requested by the owner (*Different word control*) and one in which the owner waited for the dog to perform spontaneously an identifiable action outside of a training/testing context (*Spontaneous action word control*).

#### Delayed spontaneous tests

The Spontaneous action test and the Spontaneous object-action test were also performed with delays (retention intervals) of 20 s, 1 min, and 1 h between the first identifiable spontaneous action of the dog and the repeat command. The delayed and non-delayed tests were carried out in randomized order.

In the tests with delays of 20 s and 1 min, after the dog had performed a well-identifiable action, the owner called the dog back and walked with it away from the area for the given duration of the delay. When the delay elapsed, the owner walked back to his/her initial position and gave the repeat command. In the tests with 1 h delay, after performing a potentially repeatable action, the dog was placed into its crate by the owner. The dog stayed there for the duration of the delay (all dogs were ‘crate-trained’, i.e., they were accustomed to stay and rest in their crates). This was done to ensure that the only potentially repeatable action performed in that context was the one identified before, so that, being brought in the same context again for the repeat test, the dog would have a possibility to identify and remember it (see also *Control for context* test). Moreover, taking the dog away from the view of the environment where the action was performed ensured that it could not potentially keep its mind active on the performed action by looking at some environmental stimuli. We carried out one trial per condition/delay (20 s, 1 min, 1 h), per dog.

#### Control for context

This test was carried out to test if, in the delayed spontaneous tests, the dog recognized and remembered the action previously performed, even if a delay elapsed, by being brought to the same context where it did the action before. We tested a subset of 4 dogs in an identical delayed test as the *Spontaneous action test*, with an interval of 20 seconds, but giving the repeat command in a different context from the one where they performed the action. After the dog had performed a well-identifiable action in a given area (the living room of the owner), the owner called the dog back and walked with it to a different location (in the garden: N = 3 dogs; in the terrace: N = 1 dog). When 20 seconds from the action previously performed elapsed, the owner gave the repeat command at this different place. We carried out one such trial per dog.

### Statistical analysis

Repeat success in the various tests (binary response variable) was analyzed using binomial Generalized Linear Mixed Models (R package ‘lme4’)^46^ with dog ID as random term and test condition and/or delay as fixed effects. The effects of explanatory variables were analyzed by likelihood ratio tests (LRT): we provide χ^2^ and p-values of likelihood ratio tests of models with and without the explanatory variable.

Informed consent for publication of identifying information/images in an online open-access publication has been acquired^34^.

### Ethical statement

All experiments were performed in accordance with relevant guidelines and regulations. The Institutional Committee of Eötvös Loránd University has approved the experiments of this study (N. PE/EA/2021-5/2017).

## Supplementary information

Supplementary material.

## Data Availability

Data will be available upon request.
