# Supplementary material for: Mental representation and episodic-like memory of own actions in dogs
Source: Sci Rep. 2020 Jun 26;10:10449. doi: 10.1038/s41598-020-67302-0 (PMC7320188; doi:10.1038/s41598-020-67302-0)
Supplement: Supplementary file 1 — Supplementary material. [file 41598_2020_67302_MOESM1_ESM.docx]

**Supporting information**

**Mental representation and episodic-like memory of own actions in dogs**

Claudia Fugazza^1^*, Péter Pongrácz^1^, Ákos Pogány^1^, Rita Lenkei^1^ and Ádám Miklósi^1,2^

^1^*Department of Ethology, Eötvös Loránd University, Budapest*

^2^*MTA-ELTE Comparative Ethology Research Group*

*Corresponding author: Claudia Fugazza [claudia.happydog@gmail.com](mailto:claudia.happydog@gmail.com)

**Materials and Methods**

Subjects

Ten adult family dogs of various breeds (1 female Chihuahua, 1 female miniature poodle, 1 female Shetland Sheepdog, 5 Border Collies, of which 2 males and 3 females, 2 male mixed breeds) that lived as family dogs with their owners participated in this study. Half of the subjects were already trained to imitate human actions on command ‘Do it!’ with the Do as I Do method (see below and *1* for details) when the study begun. The other 5 dogs received this training after the Repeat training – see below.

The Ethical Board of Eötvös Loránd University has revised and accepted our experimental protocol (Ref. no.: PE/EA/2021-5/2017.)

Repeat training

Dogs were trained by applying operant conditioning to repeat their own actions on request: ‘Repeat!’. The training was performed by the owners based on instructions received by the Experimenter (CF). The training started by rewarding the dogs for repeating three actions that they were already trained to perform, upon hearing the command ‘Repeat!’. Once the dogs could reliably repeat these three actions, other three familiar (i.e., already trained) actions were added. For the training, owners chose actions that their dogs could already perform reliably upon verbal request, thus the actions varied across subjects. Among others, the following actions were included in the training: spin, jump in the air, raise a paw and put paws on a stool. Once the dogs were able to repeat the six familiar actions, as assessed in the *Baseline test* (maximum three errors in a 12-trial session with the six actions used during training), they underwent the subsequent tests, including the control tests described below, in a randomized order.

Do as I Do training

The dogs were trained to imitate three familiar (i.e. already trained) actions demonstrated by their owner on command ‘Do it!’ by applying operant conditioning. Once the dogs reliably imitated these three actions, other three familiar actions were introduced in the training. The dogs proceeded to the tests once they were able to imitate these six actions as assessed in a 10-trial session with maximum two errors (Do as I Do exam – for details on the training procedure and exam see *2*).

Statistical analysis

Repeat success in the various tests (binary response variable) was analyzed using binomial Generalized Linear Mixed Models (R package ‘lme4’) (*3*) with dog ID as random term and test condition and/or delay as fixed effects. The effects of explanatory variables were analyzed by likelihood ratio tests (LRT): we provide χ^2^ and p-values of likelihood ratio tests of models with and without the explanatory variable.

Description of the tests

*Baseline repeat test*

The *Baseline test* consisted of 12 trials during which the owner asked his/her dog to perform and then repeat the six actions used for the Repeat training. This test was done to verify the success of the training process and to assess the baseline level of success of dogs repeating trained own actions.

At the beginning of each trial, the owner placed his/her dogs in front of him/her, then requested the dog to perform a predetermined action using commands known by the dog. As soon as the dog completed it, the owner placed the dog back in the starting position in front of him/her and gave the repeat command while looking straight ahead, in order not to give any inadvertent cues. After the dog had performed an action, irrespectively of whether it correctly repeated the previous one or performed something different, the test continued with the next trial, once the owner had repositioned the dog again in the starting position in front of him/her. The order of the six requested actions was semi-randomized so that every action was requested twice.

All specific tests following the *Baseline repeat test* were carried out in a randomized order for each dog.

*Clever Hans control*

The owner and another person, familiar to the dogs, stood back to back. The familiar person attracted the dogs’ attention and called the dogs in front of him/her. From this moment on, the owner closed his/her eyes and ears and sang a song loudly in order to ensure that s/he would not hear any noise potentially made by the dog. The familiar person asked the dog to perform a trained action using only visual cues (i.e. gestures). Immediately after, the familiar person touched the owner’s side with hand as an agreed signal to exchange position so that the owner now moved in front of the dog, that typically had returned to its original position after performing the requested action. The owner opened his/her eyes and ears and gave the repeat command without knowing what action the dog had previously performed. This test consisted of one trial for every dog.

All dogs repeated the previously performed action.

*Untrained actions repeat tests*

Dogs were asked to perform actions they were already trained to perform but had never been asked to repeat during the training process.

The procedure was identical to that of the *Baseline repeat test*, but the requested actions differed from those included in the Repeat training. As the already-trained actions varied from dog to dog due to their training history, the type and number of actions used in the test also varied. The number of actions on which the dogs were tested varied from 4 to 8 (mean 6.3 ± 0.6) Example of actions included: sit, touch a cone, bark, enter in the agility tunnel, jump over a hurdle, spin.

*Doing nothing*

The owner asked the dog to stay in a sitting position (‘Stay!’, i.e. not move) using cues known by the dogs and waited for 5 s. Then the owner gave the repeat command. The behaviour of the dog was recorded for the following 20 s. This test consisted of one trial for every dog.

Following the repeat command 9 dogs stayed in their position for at least 5 sec (thus repeated ‘staying’ or ‘doing nothing’). One dog lied down 4 sec after the repeat command was given.

*‘Who is acting’ test*

As the subjects were not only trained to repeat their own actions, but also to imitate human demonstrated actions with the Do as I Do method (*1*; *4*), in this test the dog was randomly asked to either repeat *its own* actions (Repeat trials: R) or to imitate actions demonstrated by *its owner* (Do it trials: D) in a single 12-trial session. The test procedure in the R-trials was the same as described above for the baseline test. In the D-trials, the owner demonstrated an action, then asked the dog to imitate it (‘Do it!’).

We also included trials in which the dog was first asked to imitate an action demonstrated by the owner and then to repeat its own actions (trials that included a Do it trial and then a Repeat trial: DR). Note that every DR trial included two actions by the dog: the imitation of the action demonstrated by the owner and then the repetition of the action just performed by itself. The order of the type of trials was semi-randomized and was the same for each dog: R, DR, R, D, R, D, DR, R, DR. The actions demonstrated or requested were those included in the baseline test.

The dogs’ performance in this test condition (88.3% successful trials) did not differ from the *Baseline*, irrespective of whether we consider the trials in which the owners issued the repeat command (Who-R trials), the Do it command (Who-D trials) or the repeat command after having imitated the owners (Who-DR trials) - LRT of Experimental condition: χ^2^_1_ = 0.88, p = 0.348; Successful trials (Fig S1).

*Spontaneous action test*

The owner sat on a bench (or chair or sofa) in a place that was familiar to the dog: its house or outdoor area, based on areas suitable for the test that were available for each dog owner and familiar for every dog. The owner was instructed to type on his/her mobile phone, thus apparently ignored the dog that had the opportunity to move freely in the area. As soon as the dog spontaneously preformed a well-identifiable action (e.g., lied down, drank water from a bowl, jumped on a sofa), the owner called the dog in front of him/her and gave the repeat command. The repeat command was always given by the owner while looking straight ahead, in order to avoid inadvertent cues (see also *Control for the ‘Clever Hans’ effect* below). Although we labelled these tests as tests with ‘no delay’, typically, there was a short delay of 5-15 sec between the action of the dog and the repeat command. This delay varied more in the spontaneous test conditions due to the fact that the dog could spontaneously move further from the owner and, consequently, it took some time to call the dog back to give the repeat command.

To prevent dogs from forming expectations of being tested by being exposed to repeated tests, we carried out one *Spontaneous action test* per dog.

*Spontaneous object-action test*

Four objects that were novel for the dog – a wooden statue in shape of an animal, a plush toy, a dog crate and a doll – were placed in a familiar area (house or outdoor area) at 50 cm from each other.

The owner approached the area with the dog unleashed and let the dog to explore freely, while the owner remained passive. As soon as the dog spontaneously preformed a well-identifiable action (e.g., touched an object with their paw, grabbed an object with their mouth), the owner called the dog and gave the repeat command while looking straight ahead. Typically, there was a short delay of 5-15 s between the action of the dog and the repeat command. We carried out one *Spontaneous object-action test* per dog.

*Spontaneous action control* and *Spontaneous object-action control*

To ensure that the dogs would not simply perform that particular action in that particular situation in any case, we carried on a control trial, where, after the dog spontaneously performed a well-identifiable action, the owner called it, but did not give the repeat command. Instead, the dog was let free again in the area for 30 s. This was done for both the *Spontaneous action test* and *Spontaneous object-action test*, so that every dog participated in two such trials overall.

None of the dogs repeated the action that they performed previously. Dogs showed various actions during this test, including sniffing on the floor, licking the owner’s face, lying down and interacting with different objects in the object test.

*Different word control and Spontaneous action word control*

The owner asked the dog to perform a trained action (similarly to the *Untrained actions repeat test*). After the performance of the action by the dog, instead of giving the repeat command, the owner spoke a word of no meaning for the dog (‘Blue’ or ‘Farfalla’), always looking straight ahead.

This control was also carried out in the *Spontaneous action test* (*Spontaneous action word control*). In this case, after the dog had spontaneously performed an identifiable action, the owner called the dog and said the word of no meaning.

This control consisted of two trials for every dog, one with the first action being requested by the owner (*Different word control*) and one in which the owner waited for the dog to perform spontaneously an identifiable action outside of a training/testing context (*Spontaneous action word control*).

In these tests none of the dogs repeated the action that they had previously performed. All dogs except for two, each in one trial, stood in their position for at least 5 sec before starting other activities after hearing meaningless word. One dog lied down and another dog looked at the owner and walked away.

*Delayed spontaneous tests*

The *Spontaneous action test* and the *Spontaneous object-action test* were also performed with delays (retention intervals) of 20 s, 1 min, and 1 h between the first identifiable spontaneous action of the dog and the repeat command. The delayed and non-delayed tests were carried out in randomized order.

In the tests with delays of 20 s and 1 min, after the dog had performed a well-identifiable action, the owner called the dog back and walked with it away from the area for the given duration of the delay. When the delay elapsed, the owner walked back to his/her initial position and gave the repeat command. In the tests with 1 h delay, after performing a potentially repeatable action, the dog was placed into its crate by the owner. The dog stayed there for the duration of the delay (all dogs were ‘crate-trained’, i.e., they were accustomed to stay and rest in their crates). This was done to ensure that the last potentially repeatable action performed in that context was the one identified before, so that, being brought in the same context again for the repeat test, the dog would have a possibility to remember it (see also *Control for context test*). Moreover, taking the dog away from the view of the environment where the action was performed, ensured that it could not potentially keep its mind active on the performed action by looking at some environmental stimuli.

We carried out one *Delayed spontaneous action test* and one *Delayed spontaneous object-action test* per delay (20 s, 1 min, 1 h), per dog.

*Control for context*

This test was carried out to test if, in the delayed spontaneous tests, the dog recognized and remembered the action previously performed, even if a delay elapsed, by being brought to the same context where it did the action before. We tested a subset of 4 dogs in an identical delayed test, with an interval of 20 seconds, but giving the repeat command in a different context from the one where they performed the action. After the dog had performed a well-identifiable action in a given area (the living room of the owner), the owner called the dog back and walked with it to a different location (in the garden: N=3 dogs; in the terrace: N=1 dog). When 20 seconds from the action previously performed elapsed, the owner gave the repeat command at this different place. We carried out one *Control for context test* per dog.

None of the dogs repeated the previously performed action.

Fig. S1.

Proportion of successfully repeated own actions or actions demonstrated by the owner in the *Baseline repeat test* (open circle) and in the *Who is acting test* (filled symbols). The graph shows responses of the 10 dogs in different test conditions: in the Do it trials (Who-D; the owner demonstrated an action and gave the ‘do it’ command); in the repeat trials (Who-R; the owner asked the dog to perform a trained action and then gave the repeat command); and in the Do it + repeat trials (Who-DR; the owner demonstrated an action, gave the ‘do it’ command and, after the dog performed an action - i.e. imitated - gave the repeat command).

**Fig. S2.**

Proportion of successfully repeated actions of 10 dogs in the different experimental conditions, including control tests. Open circles represent the *baseline condition* (BL-R), in which the dog was asked to repeat actions that were used during the Repeat training. On panel a), success rates during the *Spontaneous action test* (SA-R, black filled circles) and two corresponding control tests: the *Spontaneous action control* (when no command was issued but the dog was let free again in the test area; SA-C, red diamond) and the *Different word control* (when instead of repeat, a different word was said; SA-W, green triangle) are illustrated. On panel b), success rates during the *Spontaneous object-action test* (SOA-R, black filled circles) and the *Spontaneous action control* (when no command was issued; SOA-C, red diamond) are illustrated. In the two Spontaneous repeat tests (SA-R and SOA-R) dogs were tested with different delays (0 sec, 20 sec, 1 min, 1 h) between their spontaneous action and requesting to recall and repeat it.

**References and Notes**

1. J. Topál, R. Byrne, Á. Miklósi, V. Csányi, Reproducing human actions and action sequences: Do as I Do! in a dog. *Anim. Cogn.* ***9***, 355–367 (2006).
2. C. Fugazza, Á. Miklósi, Should old dog trainers learn new tricks? The efficiency of the Do as I do method and shaping / clicker training method to train dogs. *Appl. Anim. Behav. Sci*. ***153***, 53-61 (2014).
3. D, Bates, M. Maechler, B. Bolker, S. Walker, Fitting Linear Mixed-Effects Models Using lme4. *J. Stat. Softw.* ***67***, 1-48 (2015).
4. C. Fugazza and Á. Miklósi, Deferred imitation and declarative memory in dogs. *Anim. Cog*., ***17***, 237-247 (2014).
